# Smart Contracts and Shared Platforms in Sustainable Health Care: Systematic Review

**DOI:** 10.2196/58575

**Published:** 2025-01-31

**Authors:** Carlos Antonio Marino, Claudia Diaz Paz

**Affiliations:** 1 CENTRUM Católica Graduate Business School Pontificia Universidad Católica del Perú Lima Peru

**Keywords:** health care, smart contracts, blockchain, security, privacy, supply chain, patient centricity, system trust, stakeholders

## Abstract

**Background:**

The benefits of smart contracts (SCs) for sustainable health care are a relatively recent topic that has gathered attention given its relationship with trust and the advantages of decentralization, immutability, and traceability introduced in health care. Nevertheless, more studies need to explore the role of SCs in this sector based on the frameworks propounded in the literature that reflect business logic that has been customized, automatized, and prioritized, as well as system trust. This study addressed this lacuna.

**Objective:**

This study aimed to provide a comprehensive understanding of SCs in health care based on reviewing the frameworks propounded in the literature.

**Methods:**

A structured literature review was performed based on the PRISMA (Preferred Reporting Items for Systematic Reviews and Meta-Analyses) principles. One database—Web of Science (WoS)—was selected to avoid bias generated by database differences and data wrangling. A quantitative assessment of the studies based on machine learning and data reduction methodologies was complemented with a qualitative, in-depth, detailed review of the frameworks propounded in the literature.

**Results:**

A total of 70 studies, which constituted 18.7% (70/374) of the studies on this subject, met the selection criteria and were analyzed. A multiple correspondence analysis—with 74.44% of the inertia—produced 3 factors describing the advances in the topic. Two of them referred to the leading roles of SCs: (1) health care process enhancement and (2) assurance of patients’ privacy protection. The first role included 6 themes, and the second one included 3 themes. The third factor encompassed the technical features that improve system efficiency. The in-depth review of these 3 factors and the identification of stakeholders allowed us to characterize the system trust in health care SCs. We assessed the risk of coverage bias, and good percentages of overlap were obtained—66% (49/74) of PubMed articles were also in WoS, and 88.3% (181/205) of WoS articles also appeared in Scopus.

**Conclusions:**

This comprehensive review allows us to understand the relevance of SCs and the potentiality of their use in patient-centric health care that considers more than technical aspects. It also provides insights for further research based on specific stakeholders, locations, and behaviors.

## Introduction

### Background

New technologies have disrupted health care and imply exposure to complex scenarios. The Internet of Medical Things (IoMT) is increasingly receiving attention from practitioners and scholars to provide more alternatives for monitoring patients’ conditions; allowing access to private medical data; and obtaining secure and flawless tools to protect data from attacks that can access or steal essential information or, in some cases, produce a patient fatality [1-3]. In this new technological landscape, recent studies have highlighted that blockchain technology with smart contracts (SCs) provides the most reliable data security, cryptographic capacities, and decentralized storage and can lead to a low-cost ecosystem and sustainability in the medical setting [4,5].

The health care value chain is another research area where SCs have consistently shown effectiveness in avoiding counterfeiting and ensuring product security and safety. Implementing SCs on value chains can guarantee data provenance, eliminate unnecessary intermediaries, and provide an immutable history of transactions considering all the internal and external stakeholders. Although electronic health records (EHRs) are the most sensitive information in the SC environment, all the sensor devices are essential to provide a robust framework [6]. A health care value chain faces different challenges and aims to monitor diseases by considering real-time patient status updates. Furthermore, some visible barriers include data interoperability and communication among applications, medical devices or machines, and institutions. This increases complexity and continuously impacts costs and efficiency [7]. However, acquiring the logic of the value chain and adapting cutting-edge technologies to specific needs can save millions of lives and enhance quality of life for others.

There are different approaches to analyzing literature data. A classic methodology considers a small number of research studies to synthesize the different perspectives and angles of the topic and provide future avenues of research. In addition, there are various frameworks, such as systematic or structured reviews. Thus, a bibliometric framework considers an extensive quantity of literature by using statistical software. Multiple objectives are pursued, such as recognizing intellectual models and new trends. Our study aimed to provide a bibliometric analysis of SCs on the health care value chain and, thus, obtain a fundamental knowledge of the intellectual, social, and conceptual structure and other clustering techniques. This review’s analysis was primarily interdisciplinary, seeking to discover barriers and possible gaps in the SC domain.

### Motivation

Trust reduces complexity [8,9], manages uncertainty by compensation [8], and is an element of social capital [10]. Trust involves the truster and trustee as actors and expectations about actions in the future that could entail some risk for the trustee [11]. Trust applied to new technologies implies the transition from personal trust to system trust [8,11,12].

The literature refers to trust as a requirement of sustainable and human-centered technology [13]. Technologies, through trust, also look to fulfill social interests and be responsive. This affirmation is essential when technologies—including blockchain and SCs—are introduced in fields such as health care [11].

A blockchain is a distributed ledger [14] formed by chronologically ordered blocks [15,16]. It consists of multiple nodes connected peer to peer [15] and without a hierarchy among them [15]. Each block has an identification linked to the previous one via a reference or hash [14,15]. There is a genesis block, which is the first block of the chain, and any subsequent block also has the hash that allows for the identification of the previous one [15]. Blocks have a block header—that usually includes the hash of the last block, a time stamp, and a nonce [16]—and a payload or data with transactions [15,16]. The blockchain holds a consensus algorithm [14] that adds information to the chain [16]. Blocks are accepted or refused based on this algorithm [15]. In doing so, miners, who are a particular type of node, solve a challenge (ie, a mathematical puzzle) to verify the block and receive a reward [16] (ie, “gas”). Information cannot be modified once a block has been added to the chain. SCs are codes stored in a blockchain [17] containing transactions executed without intermediaries [14]. Thus, technologies such as blockchains and SCs promise transactions that do not require trust among parties—called trust-free, trustless [18], or trustless trust [11]—and distribute trust among the system—called distributed trust [11]. It is relevant to point out that their origins are independent despite the extensive joint use of blockchains and SCs. SCs were propounded as a transaction protocol [19], whereas blockchains uses cryptography to allow for the exchange between participants worldwide without the necessity of a central authority [20]. Blockchains and SCs look for trust development, where trust lies in the system design [18].

SCs strengthen the capabilities of a blockchain [21], aiming to (1) implement customizable business logic [22] through different functionalities [23] and (2) automatize the execution of preassigned transactions [3,24-26]. One of the most essential characteristics of SCs is that transactions are automatically executed [27]. For this, the interposition of a third party is not required [27]. This characteristic improves efficiency, accountability [28], and trust building [28,29]. SCs are transparently auditable [30] owing to their immutability. The decentralization of these systems also improves their resilience. Centralized systems represent a unique vertex of failure, a limitation that is overcome by SCs [31] deployed in blockchains [32]. Thus, SCs are especially relevant in health care as system trust generators [26,29].

### Contribution and Related Works

This study fills a gap and responds to a request in the literature. Even though SCs are relevant in health care, a sufficient assessment is necessary. Some studies characterize SCs but do not refer to health care. It is the case of the proposal by Alzhrani et al [32], which presented a characterization of some SCs in real-world systems based on a blockchain’s taxonomy. Nevertheless, this study does not focus on health care, and only 11 SCs were selected to exemplify its taxonomy. Other studies have focused on the health care sector but had a limited scope [14,33] and have not provided an in-depth characterization of SCs. Vargas and da Silva [14] assessed 3 case studies or frameworks related to SCs in the IoMT. Sookhak et al [33] limited their study to entering patient data into EHRs.

Several theoretical studies or literature reviews regarding blockchains in health care have considered or mentioned SCs in the health care domain. Nevertheless, they need to focus on providing in-depth information about the role and features of SCs. The closest one is the review by Marbouh et al [1], who inquired about the advantages of blockchains in improving patient safety. The challenges and opportunities of this technology were the starting point of the aforementioned study, which referred to the uses of blockchains in health care. It introduced SCs and some uses of blockchains. Still, these contracts were not the study’s primary goal, and it did not offer a systematic survey of the literature based on propounded frameworks.

Furthermore, Villarreal et al [29] studied blockchains in health care management systems and classified the architectural mechanisms in the literature. This study acknowledged SCs’ relevance and problems in health care, but they were not systematized and referred to one specific telemedicine case. Similarly, in the study by Arbabi et al [34], the authors surveyed studies on blockchains in health care. They acknowledged the relevance of SCs but did not review their attributes in depth and suggested further assessment of the role of SCs.

Similarly, Khatri et al [35] systematically analyzed the broad topic of health care and blockchain integration and selected 50 publications for an in-depth analysis. In their study, the authors mentioned SCs; nevertheless, they did not propound specific functionalities or roles of SCs or link them with the authors’ proposal about blockchains. Finally, McBee and Wilcox [36] studied the application of blockchains in medical imaging. Nonetheless, the literature has barely mentioned SCs to specify the origin of the name “blockchain 2.0” and its relationship with artificial intelligence (AI) [37].

Finally, Arbabi et al [34] suggested further research on SCs in health care. The authors mentioned that the potential of SCs in health care requires an in-depth assessment. In addition, Hawlitschek et al [38] propounded the development of additional research on the design of trusted interfaces. Thus, our study aimed to fill a gap in the literature—detailed in Table 1—and be responsive to its suggestions [34,38], focusing on an extensive review of SC frameworks in health care as elements that enhance trust and shift the traditional concept of trust among people to system trust, providing a characterization of it.

**Table 1:** Gaps in the literature.

Study	Health care sector	Focused on SCs^a^	Included different topics (extensive review)	Review based on propounded frameworks	The roles or characteristics of SCs were developed
Alzhrani et al [32]	No	Yes	Yes	No	Yes
Vargas and da Silva [14]	Yes	Yes	No	Yes	Yes
Sookhak et al [33]	Yes	Yes	No	No	No
Marbouh et al [1]	Yes	No	No	No	Yes
Villarreal et al [29]	Yes	No	No	No	No
Arbabi et al [34]	Yes	No	Yes	No	No
Khatri et al [35]	Yes	No	Yes	No	No
McBee and Wilcox [36]	Yes	No	No	No	No
Our study	Yes	Yes	Yes	Yes	Yes

^a^SC: smart contract.

### Research Goals

Our study aimed to provide a holistic understanding of SCs in health care. We focused on developing a structured literature review of the state-of-the-art scientific landscape of new technological advances, tendencies, and bibliometric analysis to help provide a comprehensive understanding and up-to-date overview of the recent research and outline new perspectives and future research directions.

These contributions allow this research to fill a gap in understanding and respond to the suggestions by Arbabi et al [34] and Hawlitschek et al [38]. We adopted a systematic literature review approach because it allows for the replication of this study. A quantitative bibliometric analysis followed by an in-depth qualitative review of the studies enabled us to provide a detailed standpoint of the topic.

## Methods

We used the PRISMA (Preferred Reporting Items for Systematic Reviews and Meta-Analyses) principles [37] for our systematic literature review [39]. Multimedia Appendix 1 contains the PRISMA checklist. The Web of Science (WoS) database provided the data, which were retrieved on July 4, 2023. The selection of databases for literature reviews is a topic under discussion [40], and there is no unique response. Some studies have used several databases, whereas others have been restricted to 1. Among the latter, several systematic reviews have selected WoS as their search database [39,41], including some in health care [42,43] and digital health [44]. The propounded reasons were the reputation and reliability of this source [45] and its extended use [39].

We chose to use only 1 database, WoS, because the joint use of several databases could introduce bias in our study. First, the search engines need to be unified and standardized. If we compare 2 of the most well-established databases of academic literature, WoS and Scopus [46], and one of the most relevant specialized databases in medical topics, PubMed [47], we could highlight some differences. For example, PubMed, Scopus, and WoS have the search option All Fields; nevertheless, this option encompasses different meanings for each database as they have other fields that are difficult to integrate [48] (ie, PubMed has the field Pharmacological Action that is absent from WoS; WoS has the search fields Keyword Plus and Author Keyword, whereas PubMed has the search field Word-Term, MeSH Term, and Other Terms; Scopus includes a field named References, which is absent in the other 2 databases; and WoS has the search field Topic encompassing Title, Abstract, Keyword Plus, and Author Keyword, whereas Scopus has the search field Article, Title, Abstract, Keyword. Even though they could seem the same, they are not because WoS includes KeywordPlus, which responds to a proprietary algorithm, and Scopus’s Keyword field includes both Author Keywords and Index Terms [controlled vocabulary]). Something similar happens with other well-known databases such as EBSCOhost (ie, EBSCOhost consists of a field named All Text, which is absent in WoS and other databases). The literature has highlighted the discrepancies among WoS, Scopus, and PubMed regarding different topics [49,50], such as document type [51,52], funding information [53], and subject classification [54].

The combination of information obtained from ≥2 databases also generates an additional source of bias caused by data wrangling [55]. Each database provides its search results in its format (ie, WoS uses commas [,] in some fields such as Keywords, whereas Scopus uses semicolons [;]). The different files require to be combined to assess them as one sample. This procedure could be performed manually or using an informatic tool; nevertheless, the data are always altered. For example, the order of the fields is different, and thus, we would have had to uniformize the data and the fields.

In addition to the possible bias caused by combining multiple databases as our main decision criteria to select only WoS, there is also the concern regarding coverage and overlap with other databases as different databases have unequal scopes and coverage policies [56].

There are several studies on different databases’ coverage, but they are inconclusive regarding which database could be considered the most suitable for all cases. Some databases, including Scopus, could generally encompass a higher number of articles than WoS [57,58]; nevertheless, when specific topics are searched for, the coverage could be very similar [59], and some authors have also remarked that WoS coverage depth is better [47]. WoS is a multidisciplinary database with more articles than PubMed [57], but PubMed specializes in medicine and biomedical sciences [47,49]. On the basis of the novelty of the topic under study and its relationship with other disciplines, such as engineering, using a multidisciplinary database with a broader scope was considered the most suitable option. In addition, WoS has also essential coverage in medicine, similarly to Scopus. As an example, the evaluation of the content of Scopus and WoS in the context of Norway’s scientific and scholarly publications concludes that both databases are highly and similarly comprehensive in medicine and health—with 89% of scientific publications and 87% of scholarly publications in Scopus and WoS, respectively—and natural sciences and technology—with 85% of scientific publications and 84% of scholarly publications in Scopus and WoS, respectively [60]. In addition, WoS has high levels of overlap with Scopus [58,59] and PubMed [57], which means that WoS shares an essential number of publications that are the same as those in each of the other databases (Scopus and PubMed).

Therefore, the most suitable option was to use only 1 database, WoS, for our study to avoid possible bias caused by the differences in the structures of different databases and data wrangling. In addition, WoS is a well-known and esteemed database with comprehensive coverage of the topic under study, presenting a high level of overlap with other multidisciplinary and specialized databases.

Furthermore, peer review is relevant to avoid selection bias [61]. Both authors participated in the different stages of the identification and screening process and discussed and agreed on the search terms, procedure, and screening and selection criteria before executing this process. Following previous studies [62], one of the authors (CDP) conducted a detailed review of the titles and abstracts and, when necessary, the complete texts to apply the selection criteria in the screening stage. The senior researcher (CAM) then shared and peer reviewed the results. Both authors discussed doubts and discrepancies until they reached an agreement.

Exact searches were used to identify articles to reduce the risk of bias. The terms used to refer SCs included 6 variations encompassing singular and plural for the word with and without a hyphen and the joint form, similarly to the study by Dwivedi et al [63]. The search was exact for each variation, and brackets were used. Adopting this approach did not leave the interpretation of term combinations to the subjective judgment of the researchers. The use of loose or approximate phrases was allowed for “health care.” For the term “health care,” we used an open search with a wildcard in the middle and at the end of the word (“health care*” or “health*care”). The search was Boolean. The query included the connectors OR for the same concepts and AND between different ideas considering the sense of the search and the rules of precedence of these operators in the WoS database. The query was as follows: (“smart contract” or “smart contracts” or “smart legal contract,” or “smart-contract” or “smart-contracts” or “smart legal contracts”) and (Health care* or health*care) (All Fields).

## Results

### Overview

**Figure 1:**
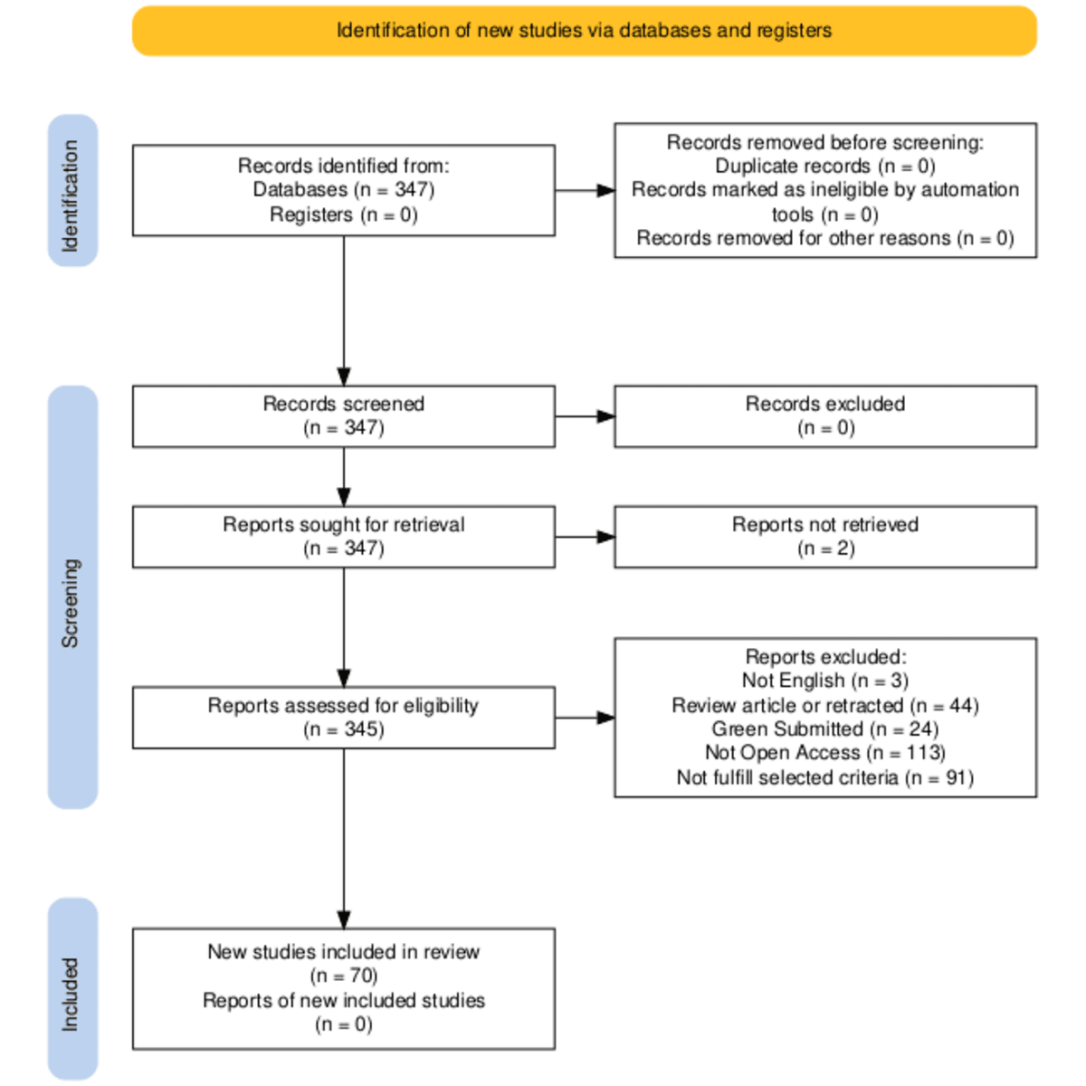
PRISMA (Preferred Reporting Items for Systematic Reviews and Meta-Analyses) framework for literature assessment.

The search produced 374 results. The exclusion criteria were based on language (only English), type of source (review article, retraction, editorial material, and retracted publications were excluded), and availability (green submitted publications were excluded, and only open access publications were included). All these restrictions were established based on WoS filters. Finally, 163 publications were assessed. The selection encompassed the following criteria. Only those publications that (1) had the health care industry as their focus, (2) included a framework (framework, system, model, prototype, or similar), and (3) incorporated details about the roles of SCs and identified SCs were included in the study. In addition, the texts of the studies by Subramanian et al [64] and Elgendy et al [65] could not be accessed. A list of the 163 papers and their assessment results based on the aforementioned criteria can be reviewed in Multimedia Appendix 2. A final sample of 70 publications was selected to be reviewed. Figure 1, which was made using the template propounded by the PRISMA organization, details the selection process [66].

### Bibliometric Assessment

We assessed the selected studies to find the main trends. The biblioshiny app and the *bibliometrix* tool (K-Synth Srl) was used in this process [67]. First, the data were screened. A total of 7% (5/70) of the studies had incomplete information about the authors’ keywords. Some techniques such as multiple correspondence analysis [68] and network analysis [50] are sensible to missing data [69]. Keeping these studies could have biased the results.

Consequently, we decided to withdraw 7% (5/70) of the articles [70-74]. Thus, we included 65 articles in the bibliometric assessment. The aforementioned 7% (5/70) of the articles were withdrawn only for these purposes; they were read in depth for reporting in the literature review section. In addition, the terms “smart contract,” “smart legal contract,” “smart contracts,” and “smart legal contracts” were signaled as synonyms where required.

The studies were published in 34 journals, and one of the journals, *IEEE Access*, published 29% (19/65) of the studies. The countries with the most citations were the United States, the United Arab Emirates, South Korea, and Egypt with >100 citations per country. Table 2 summarizes the descriptive information.

**Table 2:** Descriptive information.

Topic	Values
Sources (eg, journals and books), N	34
Documents, N	65
Annual growth rate (%)	67.03
Document age (y), mean (SD)	2.52 (1.16)
Authors, N	246
Single-authored documents (n=65), n (%)	2 (3)
International coauthorship, n (%)	32 (49)

**Figure 2:**
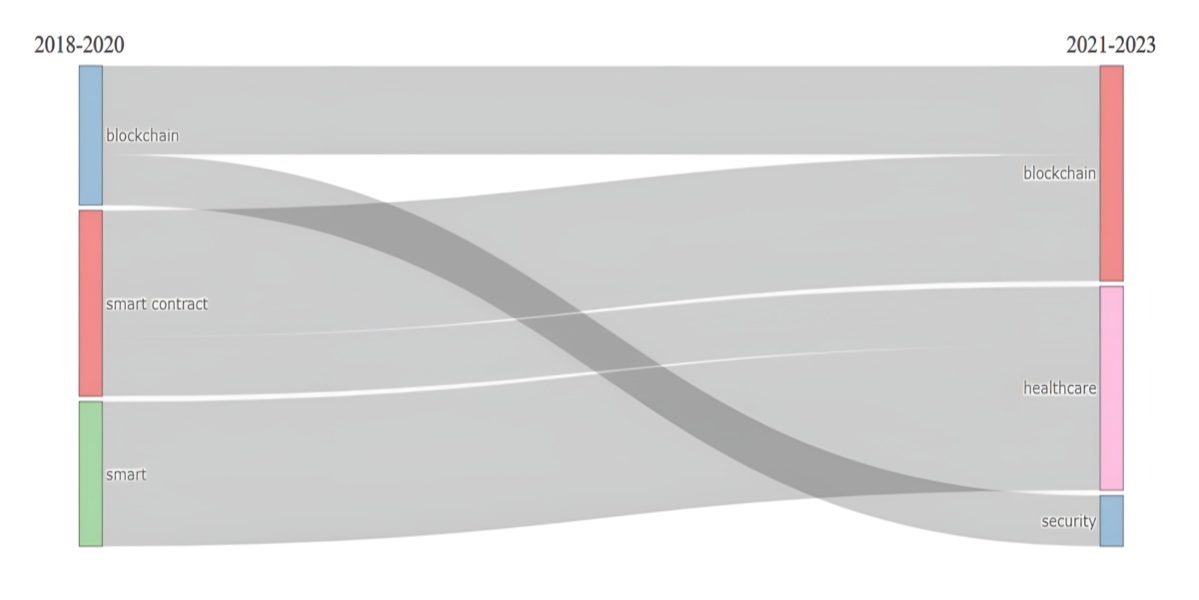
Historical evolution of the topic.

**Figure 3:**
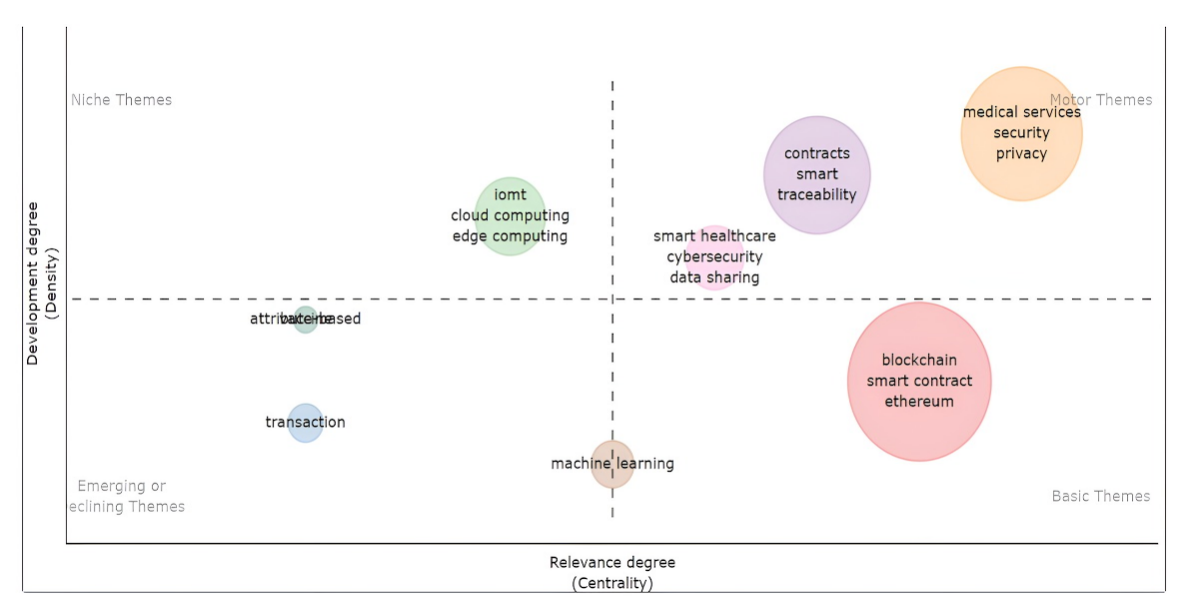
Map of subjects. IoMT: Internet of Medical Things.

The historical evolution of this topic encompasses 6 years, from 2018 to 2023. The oldest study in our group was the one by Dagher et al [75], published in 2018. Although this theme is relatively new, its historical evolution (Figure 2) reflects the relevance of COVID-19. The literature has propounded that this disease was one of the most important in recent years [76,77]. Until 2020, a total of 3 topics represented the field. After 2020, a new independent concept, *security*, emerged, and the term *health care* acquired relevance. In addition, the map of subjects organizes them into 4 groups given their significance and development (Figure 3). It shows that security, privacy, medical services, traceability, innovative health care, cybersecurity, and data sharing are the most developed and relevant topics, known as motor subjects. The IoMT constitutes a niche theme and developed topic. Cloud and edge computing are also niche themes. On the other hand, machine learning has a low level of development, which could be explained by its novelty and a medium level of relevance. This situation could reflect the first attempts to use SCs and machine learning together in the same framework.

**Figure 4:**
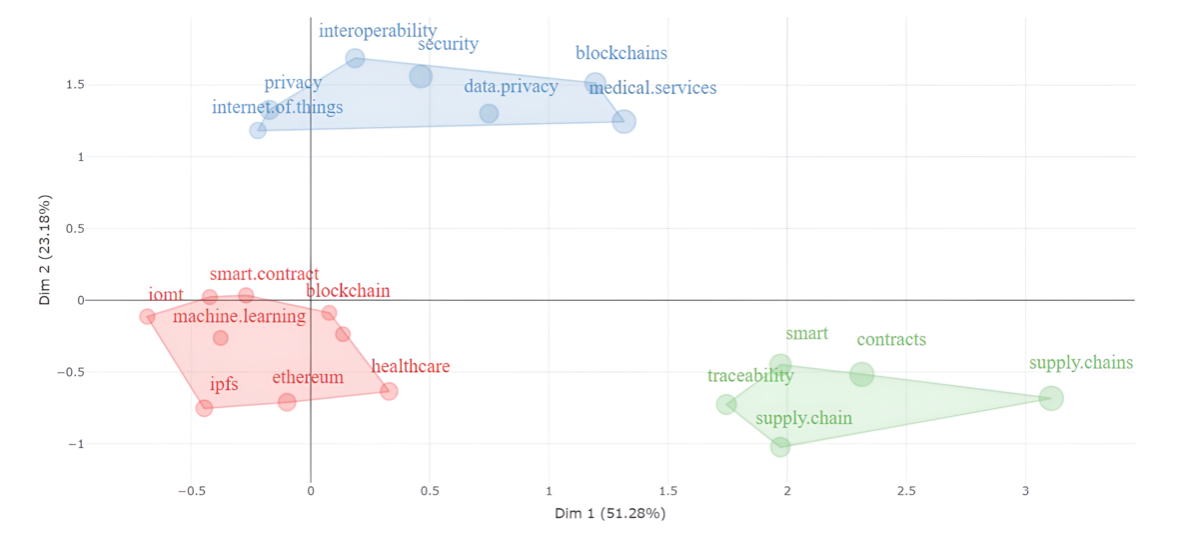
Factorial analysis. IoMT: Internet of Medical Things; IPFS: InterPlanetary File System.

**Figure 5:**
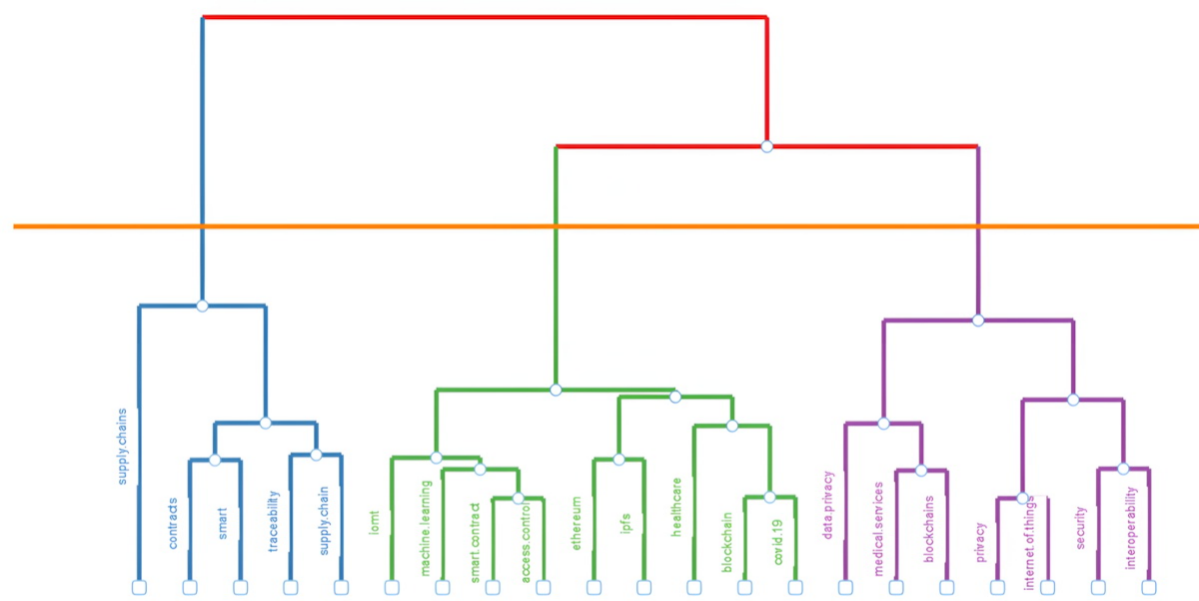
Factorial analysis tree. IoMT: Internet of Medical Things; IPFS: InterPlanetary File System.

We obtained the conceptual map and keyword cluster [67] through factor analysis, a data reduction technique. Multiple correspondence analysis was used. This technique depicts the words on a map based on a similarity measure [67]. The study yielded 3 factors in 2 dimensions (Figures 4 and 5), representing 74.44% of the variability or inertia (dimension 1: 51.28%; dimension 2: 23.18%). The factorial analysis tree supported the existence of 3 dimensions under the cutting line (Figure 5). The first factor represents the concern for the technical aspects of the topic. In this factor, Ethereum and InterPlanetary File System (IPFS) had the highest contributions (Ethereum: 3.083%; IPFS: 2.054%; together: 5.137%). The second factor concerns data privacy and security. Security, privacy, and data privacy represented the highest contributions to this factor (security: 8.191%; privacy: 4.498%; data privacy: 4.026%; together: 16.715%). Finally, the third factor is concerned with the processes themselves. Supply chain and supply chains had the highest contributions (supply chain: 5.135%; supply chains: 10.066%; together: 15.2%).

### Literature Review

#### Overview

The literature review encompassed the in-depth reading of the 70 selected articles. The factors related to processes and patients’ privacy provided an initial approach to comprehending the role of SCs in health care. A detailed review of frameworks also permitted learning of SCs’ technological characteristics, which are linked to the third factor in the quantitative stage. In addition, and following the motivation of this study, the assessment of the authors’ proposals also allowed for propounding the building elements of system trust in health care SCs.

#### Roles of SCs in Health Care

##### Overview

SCs in health care encompassed 2 main kinds of business logic, depicted in Table 3: process improvement and protecting patient privacy rights. The first business logic, process improvement, included six themes: (1) the medicine and medical equipment supply chain [3,22-24,31,78-83], (2) insurance claim attention [26,84], (3) vaccination passports and certificates [72,76,77,85], (4) clinical research [86,87], (5) emergency attention process [88,89], and (6) regulatory compliance [90,91]. The second business logic, protecting patient privacy rights, incorporated three themes: (1) patient consent management [17,71]; (2) authentication, authorization, and access [21,27,73-75,92-114]; and (3) telemedicine and eHealth care [70,74,102,115-126]. This classification aims to understand the business logic reflected in SCs based on the main goal or goals pursued in developing the framework and the most relevant stakeholders.

**Table 3:** Roles of smart contracts in health care.

Roles and themes		References
**Role 1: process improvement**		
	Medicine and medical equipment supply chain	[3,22-24,31,78-83]
	Insurance claim attention	[26,84]
	Vaccination passports and certificates	[72,76,77,85]
	Clinical research	[86,87]
	Emergency attention process	[88,89]
	Regulatory compliance	[90,91]
**Role 2: protecting patient privacy rights**		
	Patient consent management	[17,71]
	Authentication, authorization, and access	[21,27,73-75,92-114]
	Telemedicine and eHealth care	[70,74,102,115-126]

##### Role 1: Improving Health Care Processes

The studies that adopted this perspective used SCs as a lever to face problems related to processes, and their frameworks presented solutions to generate accountability and efficiency as their primary challenge. The topics encompassed in this group were medicines and medical equipment supply chain, insurance claim attention, telemedicine and eHealth, vaccination passports and certificates, clinical research, emergency attention processes, and regulatory compliance.

Medicine and medical equipment supply chains constituted the primary category of this group. The use of SCs in health supply chains [3,22,24,31,78-82] aimed to solve problems related to efficiency. These problems could be associated with tracing the supply chain stages, reducing overproduction and underconsumption, automating procurement contracts, or verifying the route of resealable returned drugs. Some studies had the specific mission of correcting malpractice, as exemplified by cases of counterfeit medicines and medical equipment [78,80,82].

Immutability, decentralization, transparency, and automatized execution of blockchains are valuable features that could solve these problems. Musamih et al [79] aimed to track all controlled drugs. SCs were required to follow all actions; thus, the actors in the supply chain would be individually accountable for their participation in the process because their activities could be tracked back. The framework included 7 actors: the controlled drug regulator, manufacturer, distributor, hospital and pharmacy, nurse station, and patient. The drug manufacturer controls the process. In total, 3 SCs were propounded in this framework: registration, production, and consumption.

One particularity of these frameworks is that several included the participation of specific government regulators, such as the Food and Drug Administration in the United States [22,31,81] and the Central Drugs Standard Control Organization in India [80], or nonspecific ones [3,79] that interact with private stakeholders. It is relevant to mention that SCs look to generate relationships without the necessity of trust among participants. Trust goes from the interpersonal level to the system level. In this case, government agencies that participate in blockchains could interact with their counterparts without the need to trust them, and vice versa.

As was previously stated, some frameworks depicted the extraordinary complexities of their stakeholders. As an example, Munasinghe and Halgamuge [82] propounded a framework that tried to uncover counterfeited COVID-19 vaccines. The authors realized that there was an international move of vaccines and then a nationwide distribution. This framework considered the ingredient provider, the vaccine manufacturer, the external company (vaccine supply), consolidation (government), the primary distribution, hospitals, clinics, pharmacies, and zone distributions as stakeholders. On the basis of these characteristics, the authors propounded 4 SCs.

Similarly, the study by Omar et al [24] depicted a particular negotiation structure of transactions. The authors applied SCs to procurement contracts negotiated by group purchasing organizations. In this context, several health providers negotiate procurement contracts with manufacturers. These health providers must pay a membership fee to the group, but they achieve better prices or loyalty rebates under this form of negotiation. The group negotiates prices with the manufacturer and determines the distributor for the entire group. This framework encompassed 5 SCs that represent the joint negotiation, the individual interests (rebates), and health care providers’ actions (purchase placement).

The frameworks related to the health supply chain could also use additional technologies in the solution, but these are the exceptions. For example, Abbas et al [78] suggested combining blockchain and machine learning techniques in the same framework. This framework sought more efficient supply chain management and provided final consumer recommendations. SCs improved the supply chain. Recommendations were propounded based on machine learning outputs. In these solutions, SCs include functionalities related to registration and production, consumption, and waste assessment.

Insurance claim frameworks include SCs to facilitate the attention of these claims, reduce [24] fraudulent activities [26,64], or improve efficiency [84]. These contracts could include government entities as stakeholders [26], health insurance providers [84], claimants, patients, and health care providers or hospitals. The process automatized by SCs includes claim submission and attention and could also encompass billing and payment processes. Unlike in supply chain studies, patients have a relevant participation in these frameworks.

SCs could also enhance the processes related to vaccination passports and vaccination certificates. These vaccination records can (1) certify the status of vaccination required to do other activities, such as traveling; (2) provide information related to possible causes of the symptoms of a patient to physicians [72]; and (3) be used in sanitary emergencies [76,77,85]. In the first and second cases, the stakeholders are mainly patients and hospitals or clinics—where the patient was vaccinated and where they request later attention based on specific symptoms—and the primary function of the propounded technology is recording and providing access to the data. The third case, referring to vaccination records in emergencies such as the COVID-19 pandemic, involves more complex frameworks, including generating certificates and providing access to them to several stakeholders. These frameworks also consider the international movement of people and countries’ requirements regarding vaccination [77,85]. Stakeholders include hospitals, vaccination centers, and governmental entities—ministries of health and foreign affairs [77,85].

Clinical research can use SCs for sharing and aggregating health care–related data because SCs provide secure storage and querying while protecting privacy in managing the data [86]. Both features are particularly relevant for research because they could promote collaboration among entities and data aggregation. Increasing the amount of data could also improve the generalization of studies [86]. Hospitals, as stakeholders, could act together to develop research activities [87]. For example, Kuo and Pham [87] introduced a framework allowing different hospitals to assess data during the COVID-19 pandemic. This inquiry encompassed 13 hospitals that acted as a federation, which means that these institutions were required to share information and obtain, in some cases, a global aggregate.

The provision of attention to patients in an emergency context can also benefit from this technology [88,89]. For example, Ksibi et al [88] suggested using SCs to manage processes related to an emergency caused by a car crash. This framework allowed emergency vehicles to improve communication with the cars involved in the accident and with hospital emergency services. Peyvandi et al [89] established a framework that supported the prediction of diagnoses—or computer-aided diagnosis—in an emergency. Machine learning technology was used to forecast a diagnosis, whereas SCs preserved patients’ privacy and granted access to the data. The stakeholders are emergency units that attend hospital emergency services.

Regulatory compliance could be enhanced using SCs [90,91], detecting and flagging privacy regulation violations. SCs allow patients to select their data privacy preferences, record events, and verify compliance with regulations. Regulatory compliance could also be enhanced using edge computing [90]. In these appraisals, the stakeholders are patients, health care providers—including hospitals—and clinical researchers. Governmental agencies are not included as the perspective of these frameworks is not enforcement but private compliance.

It is pertinent to note that these frameworks mentioned the United States [22,31,81] and India [80] through their regulators. They also referenced other regions such as Southeast Asia [85]. The studies were also concerned with complying with the regulations of the European Union [91] and the United States [90]. In addition, institutions located in the United Kingdom [87] and the United States [78,86] provided datasets used to test proposals.

##### Role 2: Improving Patient Privacy Protection

A new paradigm has influenced patient data management: patient centrality [15,92-94]. Patients are acknowledged as owners and managers of their health data [92]. Moreover, Jadav et al [115] have also alluded to a new reality in this field characterized by machine-centric interaction [92], where technology—especially the IoMT—provides different solutions for the health care sector. Both new complementary paradigms, patient centrality and machine-centric interactions, guide data management.

Data fragmentation [17], data breaches [86,116], compliance with regulations [115], cross-organizational coordination [95], and interoperability [96] are some challenges that the frameworks deal with. The stakeholders are patients and health facilities—including hospitals, clinics, physicians, laboratories, and researchers. Hospitals and clinics could act independently or as a consortium. Moreover, the blockchain could also act as a federation. Hashim et al [96] highlighted the problems that arise from the interoperability of independent blockchains and proposed a solution based on 3 SCs (search, global, and local). It is also relevant to mention that one study applied its framework in an animal health care service [97].

This role includes three main topics: (1) patient consent management; (2) authentication, authorization, and access; and (3) eHealth care. The patient centrality paradigm is reflected in patient consent management, which includes the possibility of patients defining viewer authorizations. For example, El Majdoubi et al [71] propounded a framework in which patients could manage their health data privacy preferences. A privacy agreement and enforcement were automatically settled if patients’ preferences coincided with provider policies and privacy law. A privacy offer was published; this privacy offer could become a privacy agreement if it was accepted. After that, the system tracked the execution of this agreement. Thus, compliance with legal requirements and the stakeholders’ preferences was ensured. In addition, this framework encompassed 3 levels of privacy. The first one, or P0, included data that could be viewed only by patients. The second one, or P1, referred to data that health care providers could also access. Finally, the third one, or P2, contained publicly available data [76].

Authentication, authorization, and access are central topics in patient privacy protection. SCs in these frameworks deploy different functions. They manage patients’ consent, transfer and share data, search for patients, administer registration (add new users, view users, delete users, and create accounts), actualize data (add and update information), request access permission, restrict access, store data, provide search functions, and establish viewership criteria. The complexity of SC functions depends on the framework scope. The main stakeholders are hospitals and patients, as well as insurance companies and governmental agencies [98].

In addition, some frameworks use additional protocols or technologies to protect patient data privacy better. Saidi et al [99] used the self-sovereign identity (SSI) model. SSI aims to prove the identity in a digital environment, and a verifiable credential and a decentralized identifier underpin it. SSI has given rise to the SSI-based access control that allows for separate authentication, which is decentralized, and authorization, which is centralized. The SC—policy decision SC—provides efficiency and security to role assignments. In addition, this framework introduced an adaptive access control policy for emergencies. Other authors used ciphertext-policy attribute-based encryption (CP-ABE) [27,100] and attribute-based access control [95]. CP-ABE [81] and attribute-based access control determine access based on attributes; nevertheless, the first one, CP-ABE, offers a higher degree of granularity and is related to data encryption [100]. Biometric technology [20] such as fingerprints was also included in access control [21]. The use of ring signature and stealth address [20,87] to improve security was also considered [101]. Ring signature can hide the sender’s identity, keeping the transaction safe because the receiver has the elements to verify the transaction authenticity [101]. Meanwhile, a stealth address maintains the anonymity of the sender’s address, creating a 1-time address [101].

eHealth care and telemedicine represent a particular case, where SCs are mainly recommended for granting patient data access in a secure environment. Commonly, these frameworks are mentioned together in telemedicine, the IoMT, and eHealth [70,90,117], including wearable sensors, smart devices [118], and ambient intelligence [119]. Telemedicine faces several challenges, including interoperability, incorrect diagnosis, and unfavorable perceptions. These perceptions are grounded on the fact that they are not the same as physical environments [120]. In telemedicine, the frameworks use SCs to generate secure transactions and automate participant communication [120]. In doing so, the frameworks could encompass laboratory test results [90], payment, and drug delivery, among other things [102,118,120]. The stakeholders include the usual ones—patients, hospitals, physicians, insurers, medical research organizations, and laboratories—and special ones, such as a telemedicine center [120] or diagnostic center [102] and federated hospital clouds [90]. Javed et al [2] referred to the health care regulator in their framework and gave the regulator control of the blockchain.

Telemedicine and eHealth care are concerned with malicious nodes and use additional technologies to deal with this problem or detect health abnormalities. Puri et al [70] suggested using SCs and AI to identify malicious nodes and security breaches. Similarly, Jadav et al [115] recommended including AI, specifically recurrent neural networks, to ensure data integrity. Neural networks train data to detect attacks and review data before storing them. Baiju et al [121] relied on machine learning (logistic regression) to anticipate abnormalities in the stored data. Dhasarathan et al [116] adopted a supervised machine learning approach to monitor risk factors in data transmission. In addition, Masud et al [119] suggested a framework that uses biometrics, among other technologies. The use of edge computing [122] and fog-cloud computers was also suggested [74,123,124].

In addition, Shaikh et al [125] suggested a framework that aimed to transform medical data into wisdom. In this framework, data collected from patients were cleaned and transformed into information, which was evaluated and converted into knowledge. Finally, metadata were extracted and converted into wisdom that could be used in medical research. The stakeholders were patients, physicians, data analysts, and knowledge managers. SCs provided registration advantages, data privacy customization, and exchange policies in this proposal. Abou-Nassar et al [126] were also concerned about the knowledge management and interoperability of IoMT devices related to semantic differences. The authors proposed an ontology model to achieve a higher level of trust.

It is relevant to mention that, without prejudice to their generalization capability, several studies obtained their testing dataset from institutions in the United States [17,89,94,100,101,103,115] and South Korea [127]. Some studies dealt with regulatory privacy concerns in the United States (Health Insurance Portability and Accountability Act; HIPAA [21,75]) and Europe (General Data Protection Regulation [95]). Finally, 2 frameworks were tailored for specific countries: the United Arab Emirates [98] and Italy [104].

#### Technical Characteristics That Provide Efficiency to Frameworks That Incorporate SCs in Health Care

The frameworks that propound the use of SCs face an essential challenge regarding their efficiency, requiring specific technical strategies to optimize their use [128]. The literature [28] mentioned limited data storage and inefficient execution as problems of SCs [129]. One of the strategies used to solve data storage problems is in-chain and off-chain data storage. Only the most relevant information is kept in the chain; additional information is sent to another source. IPFS is the preferred solution for maintaining data off the chain. This system optimizes resources because access to the data requires only the hash generated when the data are stored [105].

Some frameworks had an essential level of complexity and were required to preserve a critical quantity of data. Doing it in the blockchain could turn the system into an inefficient one. For example, Debe et al [22] propounded a framework for tracking resalable returned drugs that included several stakeholders and SCs. The medicines were produced by manufacturers in lots. These manufacturers were also required to share images of the package. Storing these images in the blockchain would be costly and inefficient. Thus, they were stored off the chain in the IPFS. Rai [80] suggested a similar solution, which also proposed keeping a lot of drug images in the IPFS.

The IPFS was also used in frameworks focused on patient data privacy and security. For example, Azbeg et al [105] propounded that only hash data should be stored in the chain. The IPFS preserved additional data as the off-chain storage, and Hussien et al [100] introduced a procedure to encrypt medical data before their storage in the IPFS, extending the CP-ABE and comprising searchable symmetric encryption [86].

Another strategy proposed to optimize resources was the specialization of SCs. SCs contain functions deployed when they are called. Under this strategy, the different parts that the framework requires are organized in several specialized SCs that could be individually deployed when needed, optimizing the resources. The quantity of SCs and the functions included in each could vary, and they are related to the complexity of the objectives that the framework aims to achieve. Frameworks based on 1 SC were the exception (ie, the study by Peyvandi et al [89], who proposed a single SC for patient data sharing).

In addition, the specialization of nodes was also propounded [78,97]. For example, Abbas et al [78] established a framework to manage medicine supply chain management and a recommendation system to avoid counterfeit drugs. This framework leveraged the attributes of SCs using machine learning. The SCs had an execution rate that was lower than desirable. The solution was to deploy them only on specified nodes, and only some of them—called endorsers—could validate the transactions. This solution enhanced the efficiency of the system.

The frameworks’ efficiency and optimization are reflected in their cost analysis. The Ethereum platform provides the cost of gas or ether for function deployment. This cost can be converted to a specific national currency. Gas is relevant because it incentivizes miners to work [24] and protects them from distributed denial of service attacks [82]. Omar et al [24] and Chen et al [72] reported the cost based on a low average and fast execution in this platform converted to US dollars. The Hyperledger Fabric platform does not include ether; nevertheless, a computational cost can be calculated and compared. Munasinghe and Halgamuge [82], for example, calculated and compared the cost of their framework developed in Hyperledger Fabric based on previous inquiries.

It is relevant to mention that the frameworks used extended cryptography mechanisms of blockchains, such as hash functions, digital signatures, public-private keys, and Merkle trees. Ethereum and Hyperledger Fabric were the preferred platforms. The frameworks used the features and facilities provided for these platforms (ie, the programming language Solidity in Ethereum). The different types of blockchain were represented in the frameworks. There were private blockchains (ie, the study by Omar et al [23]), public blockchains (ie, the study by Debe et al [22]), and consortiums (ie, the study by Mackey et al [26]). A detailed review of this specific topic can be found in the study by Sookhak et al [33]. Ganache, Truffle, and MetaMask were frequently used [26,31]. The SCs were commonly tested in Remix IDE [24,31].

#### Characterization of System Trust in Health Care SCs

**Figure 6:**
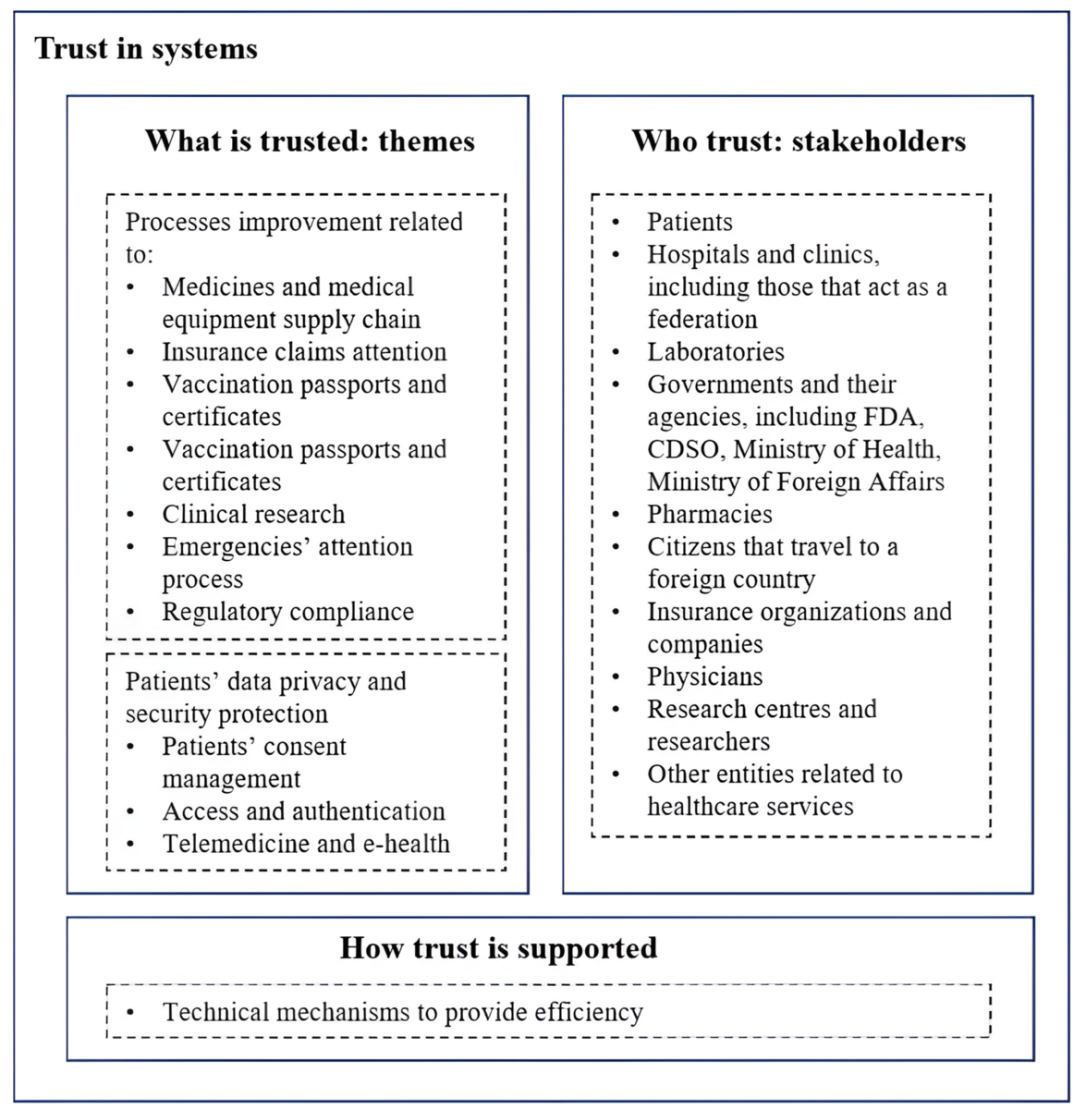
Characterization of system trust in health care smart contracts. CDSCO: Central Drugs Standard Control Organization; FDA: Food and Drug Administration.

SCs imply a transition from trust in people to trust in systems [8,11,12] where people interact without needing to trust each other or a central authority. On the basis of the previously exposed findings, we characterized system trust in health care SCs. The object of trust, or what is trusted, is represented by the 2 roles identified and the subjects they encompass. The stakeholders that interact without the necessity of a central authority are the subjects that are trusting and represent whom they trust. Finally, the technical strategies that these frameworks adopt support the trust in this system. Figure 6 summarizes these elements.

### Coverage Bias Assessment

#### Overview

This study used a single database, WoS, to avoid bias caused by the differences in database structure and data wrangling considering that the literature supports WoS’s acceptable coverage and overlap with other databases. Nevertheless, assessing possible bias caused by the difference in coverage among different databases was considered relevant. Thus, subsequently, in our study, a comparison of coverage and overlap between WoS and other databases was conducted. The selected databases were (1) a multidisciplinary one of extended use, Scopus; and (2) a specialized one, PubMed.

The procedure had 2 stages. The first conducted a comparison between WoS and Scopus and between WoS and PubMed regarding their coverage and overlap. In this stage, the queries were evaluated to make them as similar as possible. The raw results on WoS were compared to identify the overlap between studies in both WoS and Scopus or both WoS and PubMed.

The second stage asked whether the articles published in Scopus or PubMed but not in WoS could provide relevant additional information to our literature review. Thus, we selected those studies that were only in Scopus or PubMed and were published until June 2023. They were then evaluated based on the 3 selection criteria of our research. For such purposes, the titles and abstracts and, in case of doubt, the full texts were reviewed by one of the researchers. The results were then discussed between the researchers, and the doubts and discrepancies were resolved. Third, the selected studies were read and compared with the literature review results. The searches for comparison purposes in PubMed, Scopus, and WoS were conducted on June 5, 2024.

#### Coverage Bias Between Multidisciplinary Databases: WoS and Scopus

The starting point of the comparison between WoS and Scopus was to select the most suitable field in both databases to conduct the search as All Fields was not adequate. The fields Topic in WoS and Article, Title, Abstract, Keyword in Scopus were close enough to our purposes. In addition, the difference between a search of All Fields and Topic was only 8 articles. Restrictions regarding language (only English), access, and type of article were included. The queries were as follows: Refine results for (“smart contract” or “smart-contract” or “smart contracts” or “smart legal contract” or “smart-contracts” or “smart legal contracts”) (Topic) AND (Healthcare* or health*care) (Topic) and English (Languages) and Review Article or Retracted Publication or Retraction or Editorial Material (Exclude – Document Types) and Green Submitted (Exclude – Open Access) and All Open Access (Open Access) for WoS and ( TITLE-ABS-KEY ( “smart contract” OR “smart-contract” OR “smart contracts” OR “smart legal contract” OR “smart-contracts” OR “smart legal contracts” ) AND TITLE-ABS-KEY ( ( healthcare* OR health*care ) ) ) AND ( LIMIT-TO ( LANGUAGE, “English” ) ) AND ( LIMIT-TO ( OA, “all” ) ) AND ( LIMIT-TO ( DOCTYPE, “ar” ) ) for Scopus.

These searches produced 205 and 270 documents for WoS and Scopus, respectively. The results were then compared based on the digital object identifiers to obtain the level of overlap between both databases. The databases shared 181 documents, which represents an 88.3% (181/205) coincidence for WoS and a 67% (181/270) coincidence for Scopus. Notably, these percentages are higher than those obtained in other systematic reviews that also used WoS as their search engine. Maia et al [130] obtained a 61.4% (167/272) and 56.1% (213/380) overlap for the WoS and Scopus results, respectively.

Despite the high level of overlap, the next question was whether Scopus’s additional documents could provide relevant qualitative information for our study. Thus, in the second stage, we selected the Scopus documents published before July 2023 because we conducted the search for our review on July 4, 2023, and 71 papers were obtained. We then applied the selection criteria used in our study. Finally, a list of 15 articles was retrieved and reviewed in depth. Most of them referred to data exchange and EHRs and could be included in the second role of SCs related to privacy and security [65,131-140]. The additional ones could be included in the first role of SCs related to process improvement [141-144].

#### Coverage Bias Between Multidisciplinary and Specialized Databases: WoS and PubMed

A procedure similar to the previously detailed one was performed between WoS and PubMed. PubMed used the field Title/Abstract. Filters for language—only English—and text availability—free full text—were then applied. The WoS query was the one previously detailed, and the PubMed query was as follows: ((“smart contract”[Title/Abstract] OR “smart-contract”[Title/Abstract] OR “smart contracts”[Title/Abstract] OR “smart legal contract”[Title/Abstract] OR “smart-contracts”[Title/Abstract] OR “smart legal contracts”[Title/Abstract])) AND ((Healthcare*[Title/Abstract] OR health*care[Title/Abstract])).

These searches produced 77 and 205 documents for PubMed and WoS, respectively. A total of 4 PubMed documents did not have a digital object identifier and were not considered in the comparison with the remaining 74 papers. A total of 49 of those documents were also included in WoS, which represents a 66% (49/74) overlap with PubMed. On the basis of previous studies [130], this percentage can be considered satisfactory.

The second step started with selecting PubMed documents published until June 2023. The result was 11 documents. The 3 selection criteria were applied to those documents, and only 9% (1/11) fulfilled them [145]. This study [145] dealt with privacy preservation and can be included in the second role of SCs.

## Discussion

### Principal Findings

This study aimed to provide an extensive understanding of SCs in health care through a comprehensive review of their roles and characteristics based on the frameworks developed in the literature. In doing so, this study fills a gap in the literature [34]. SCs are code or short programs whose output is a transaction [15] that is produced automatically when certain previously established conditions are met. These programs reflect the business logic [15] and provide flexibility (requirements can be introduced) and efficiency (their execution does not need additional enforcement) to a blockchain. The extensive review of frameworks offered insights into the business logic that was prioritized.

A quantitative bibliometric assessment highlighted the topic’s novelty and importance, including subjects with high relevance and development. In addition, 3 factors were identified. These factors guided the in-depth review of each framework. Two roles of SCs were found—(1) process improvement, which encompassed 6 topics; and (2) patient data privacy enhancement, which encompassed 3 topics—and technical strategies and features that provide efficiency were found. These results provide more detailed information than previous inquiries, which have mentioned only this technology’s use for medical devices, prescription tracking, remote patient monitoring, counterfeit drugs, EHRs, and incident report systems [1]. In addition, several stakeholders were identified.

This study acknowledged the relevance of technical topics. Previous studies have provided taxonomies based on technological features that consider the blockchain type, ledger type, consensus, identification, authentication and authorization, and EHR storage and features [33]. This study provided a new perspective based on the features selected by the literature that provide efficiency to systems.

The studies on trust and health care had different scopes. Some authors focused on trust in the system [146,147]. Others considered trust among various actors in this system, such as physicians and patients [25]. Independently of the orientation, the main proposal was that trust is relevant for accomplishing health care goals. The quantitative and qualitative review findings characterized system trust in health care SCs, which considered systems [146,147] and actors [25], through what is trusted, who trusts, and how trust is supported.

Finally, this study revealed different levels of impact on the concerns of multiple health care stakeholders. Individuals’ rights—such as privacy [17,71], mobility [72,76,77,85], or health [88,89]—can be better protected. Physicians and researchers can use enhanced access to traceable and secure data when required [21,105]. SCs offer traceability, immutability, transparency, decentralization, automatization, and security to the different suppliers related to health care—such as hospitals, clinics, laboratories, research centers, and pharmacies—for their processes [16]. Governments can also obtain benefits from SCs. SCs facilitate private compliance with governmental regulations [90,91], preventing crime such as counterfeit drugs. In addition, SCs can facilitate the coordination of processes that require governmental intervention [22,31,81]. This study highlights new technologies, such as consensus and cryptographic methods, to address data security and privacy concerns. Second, this study details the different efforts to make potential solutions scalable and provide them to policyholders. Finally, this study shows the complexity of the health care systems, which is crucial for understanding the definition of multiple agreements among various involved stakeholders.

### Strengths and Limitations of This Review

The strength of this review lies in its systematicity and comprehensiveness. This review evaluated studies that included SCs in health care in their frameworks. We applied several procedures to reduce the risk of selection bias. The criteria used in the selection process tried to avoid researchers’ subjectivity, provide transparency, and allow for the review’s replicability [148]. The PRISMA principles were followed, a peer review procedure was contemplated, 1 database was selected, and its coverage and overlap with 2 databases—a specialized and a multidisciplinary one—was assessed. Moreover, quantitative bibliometric techniques were guided by and complemented with an in-depth and detailed literature review.

Even so, the selection of articles had 2 significant limitations. First, the different academic databases needed to be unified or standardized. Indeed, we had to decide between possible bias caused by the differences between databases and data wrangling or selecting 1 database and assessing its coverage and overlap. Our decision was the latter, but we acknowledge that it entails a limitation. Second, our study included only open access documents because they were relevant to guarantee the review of this study and ensure our research’s transparency.

### Conclusions and Further Research

#### Overview

The theme of SCs in health care is not only novel but also relevant. SCs reflect the business logic into the blockchain. Using SCs is advisable to enhance access to health records with advanced tiers of security and privacy. They can also solve other problems requiring security and traceability, such as counterfeit drugs. SCs provide benefits to several stakeholders, both individual (ie, patients, researchers, and physicians) and institutional (ie, hospitals, clinics, and governments), who interact in the context of the themes that SCs cover without the need to know each other or having a central authority or intermediaries, supported by the technical mechanisms that provide efficiency to the processes. SCs have limitations, such as data storage and use of resources [129], but techniques have emerged to deal with these issues [28].

The literature review provided some topics that could be considered in further research regarding specific stakeholders, locations, behaviors, and issues.

#### Specific Stakeholders

Although the relevance of children’s health data sharing is essential, there is a lack of studies that have dealt with the particular characteristics of this topic. The concept of patient centrality can be challenging in cases involving children and teenagers with limited control over their information. Moreover, no study has analyzed the characteristics of health care privacy protection for minors. The closest one is the study by Dagher et al [75], which acknowledged possible special conditions of the owner, such as the existence of a parent or guardian [84].

#### Specific Locations

SCs’ advantages can help solve problems related to low- and middle-income countries. Nevertheless, only some studies focused on solving the particular necessities of low- and middle-income countries using SCs. Abbas et al [78] referred to counterfeit drugs as an extended problem in countries with weak economies, and Rai [80] designed their framework for the specific case of drug traceability in India.

#### Specific Behaviors

Hawlitschek et al [38] highlighted the relevance of considering fundamental interactions among people and systems. The authors named these interactions behavioral layers. It is necessary to decode these real-world behaviors so that they can be modeled into systems. This affirmation has an extended application to the SC because it oversees the deployment of business logic in the chain. Nevertheless, evidence must be provided regarding how these real-world behaviors are deciphered and converted into systems.

#### Specific Topics

First, the internet is a system requirement [88] and is considered an element that provides ease and simplicity [75,88,106] to SCs. Nevertheless, only a few aspects are known about the proper relationship between the quality of internet connectivity and the possible expansion of the use of SCs. Second, the cost of transactions is an existing health care problem [20]. SCs allow for the automation and optimization of these transactions and save costs [74,123]. Indeed, some studies provided a cost analysis based on the computational cost (gas cost) [75] and indicated the execution cost in a specific currency [24,79,91]; a qualitative cost comparison between a blockchain-based solution and a traditional one was made [85], and the execution cost based on workflow was also assessed [123]. Nevertheless, contrary to other industries [149-151], there is a lack of studies evaluating cost as a factor in the adoption of SCs in health care.

## Appendix

Multimedia Appendix 1 PRISMA (Preferred Reporting Items for Systematic Reviews and Meta-Analyses) checklist.

Multimedia Appendix 2 List of 163 articles.

## Abbreviations

AI: artificial intelligence
CP-ABE: ciphertext-policy attribute-based encryption
EHR: electronic health record
HIPAA: Health Insurance Portability and Accountability Act
IoMT: Internet of Medical Things
IPFS: InterPlanetary File System
PRISMA: Preferred Reporting Items for Systematic Reviews and Meta-Analyses
SC: smart contract
SSI: self-sovereign identity
WoS: Web of Science
