# Supplementary material for: Smart Contracts and Shared Platforms in Sustainable Health Care: Systematic Review
Source: JMIR Med Inform. 2025 Jan 31;13:e58575. doi: 10.2196/58575 (PMC11874880; doi:10.2196/58575)
Supplement: Multimedia Appendix 2 [file medinform_v13i1e58575_app2.docx]

### Multimedia Appendix

https://github.com/ClaudiaDiazPaz/SC-in-HC/blob/main/Full%20List%20of%20163%20articles%20-%20for%20GitHub.xlsx
